# Wadi el-Sheikh: A new archaeological investigation of ancient Egyptian chert mines

**DOI:** 10.1371/journal.pone.0170840

**Published:** 2017-02-02

**Authors:** E. Christiana Köhler, Elizabeth Hart, Michael Klaunzer

**Affiliations:** 1 Institute for Egyptology, The University of Vienna, Vienna, Austria; 2 Department of Anthropology, University of Virginia, Charlottesville, Virginia, United States of America; 3 Deutsches Bergbau-Museum Bochum, Bochum, Germany; Max Planck Institute for the Science of Human History, GERMANY

## Abstract

This article provides an overview of the first results from archaeological investigations at Wadi el-Sheikh in Egypt by the University of Vienna Middle Egypt Project. Chert was an important raw material used to produce tools, implements and jewelry in ancient times. Wadi el-Sheikh was exploited over thousands of years as it was probably the most important source of chert in Pharaonic civilization. The results of our new investigations that involved surveys and test excavations indicate the presence of large scale mining activities in the first half of the 3rd Millennium B.C.E. which allow for detailed insights into the amount of raw material extracted, the mining methods used and the lithic products manufactured in this area. These aspects are contextualized on the background of ancient Egyptian state-organized resource acquisition strategies and economy.

## Introduction

Wadi el-Sheikh in Egypt is a major source for the acquisition of chert, often also referred to as flint or silex. This is the raw material used primarily for the manufacture of tools and implements during the prehistoric and Pharaonic periods at least until the end of the 2nd Millennium B.C.E., i.e. over many thousands of years, before metal fully replaced stone on a larger scale. Wadi el-Sheikh (in the following also 'the Wadi' as *wadi* is the Arabic word for 'valley') was first discovered by the British officer-cum-adventurer Haywood W. Seton-Karr in 1896 who conducted two expeditions and subsequently distributed his numerous surface finds to museum collections world-wide. He already recognized the importance of this discovery for ancient chert mining [1]. A few archaeologists have visited and briefly surveyed the Wadi in the following century; each time, they essentially concurred with Seton-Karr on the significance of this area [2, 3, 4, 5], but no systematic archaeological investigation has ever taken place. Also, those few who did visit the Wadi engaged in relatively superficial examinations of limited areas. While being valuable studies in their own right, such research has only ever provided a snap shot of activities that have taken place there [6] and have not done justice to the size and complexity of this area as a whole. This changed recently when the University of Vienna Middle Egypt Project set out to survey substantial portions, excavate select areas and to design a long-term research project for the Wadi with the aim to scientifically investigate this area using a thorough and systematic approach. This article covers fieldwork conducted over three brief seasons of different research activities during 2014 and 2015. As a result of this recent research, it is now possible to provide a more comprehensive assessment of the archaeological evidence and to demonstrate its great potential for archaeological research on ancient Egyptian resource acquisition, economy and lithic technology.

## Location, setting and condition of the mining area

**Fig 1:**
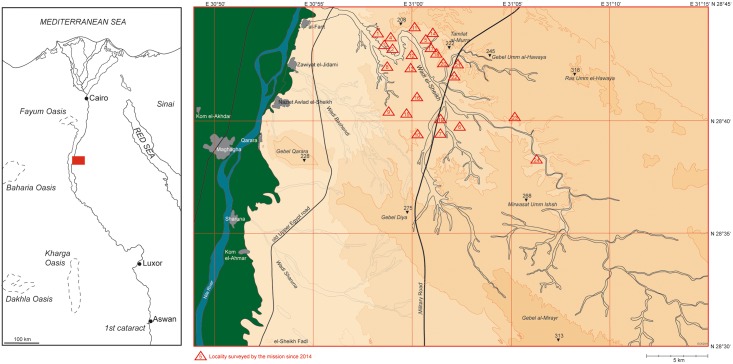
Map of Egypt and of Wadi el-Sheikh. The red rectangle on the left shows the location of Wadi el-Sheikh in Middle Egypt. The map on the right shows Wadi el-Sheikh and its surroundings; red triangles indicate localities surveyed by our mission until 2015 (Images E. C. Köhler).

Wadi el-Sheikh is located at the northern edge of the Egyptian governorate el-Minya in the Eastern Desert mountains connected to the Nile Valley, approximately 150 km south of Cairo. It is an arid desert valley, which starts in a multitude of small, winding tributaries some 35 km southeast of the modern villages of el-Fant and el-Hibe. After about 20 km along its path it unites into one single and relatively broad, yet equally meandering main valley that eventually drains into the alluvial plain of the Nile (Fig 1). As far as our research allows us to state at this time, there is evidence for chert exploitation along the entire length and on both sides of the Wadi, i.e. covering an area of at least 120 km^2^, although incalculable areas are yet to be explored as the project continues. There is also evidence for the ancient extraction of other materials including silicified limestone, salt and gypsum, but to a lesser extent. Also, local inhabitants of the area report that small scale salt mining occurred until the last century. The mineral deposits that were of interest to ancient miners are located mainly on mid-level terraces of the Wadi (approximately 70–200 m above sea level) where they are embedded in Eocene limestone formations [7]. Depending on the terrain and geology, the chert deposits can be found either naturally exposed along the edges of the terraces or entirely covered by other geological formations and thus buried deep in the mountains. The main kind of chert found in Wadi el-Sheikh is tabular in form, but sometimes it also occurs in the form of flat tabular nodules. The mineral deposits are vast as is shown by the locations of identified mining activities throughout the Wadi (Fig 1). To date we have surveyed two dozen localities (= L) where mining activities have taken place, which together cover almost 11 million m^2^, the largest single locality being over 2 million m^2^ (L6: 2.169 km^2^) in area.

Apart from the enormous dimensions of the mining areas in the Wadi, they are also difficult to access, which is probably one of the reasons why the Wadi has resisted systematic investigation until recent times. There are two ways to approach the Wadi today; either from its mouth near the edge of the desert where the old Upper Egypt desert road passes through, or from the so-called Military Road, a recently built modern highway that crosses the Wadi further east, about midway. In each case it is necessary to use a strong 4WD vehicle to make fast progress while driving along the sandy Wadi bed or ascending the slopes to directly access the terraces where mining activities have taken place. In some areas, for example where the slopes are too steep or deeply dissected by smaller gullies, even a 4WD car is not suitable and it is then necessary to walk and climb by foot.

**Fig 2:**
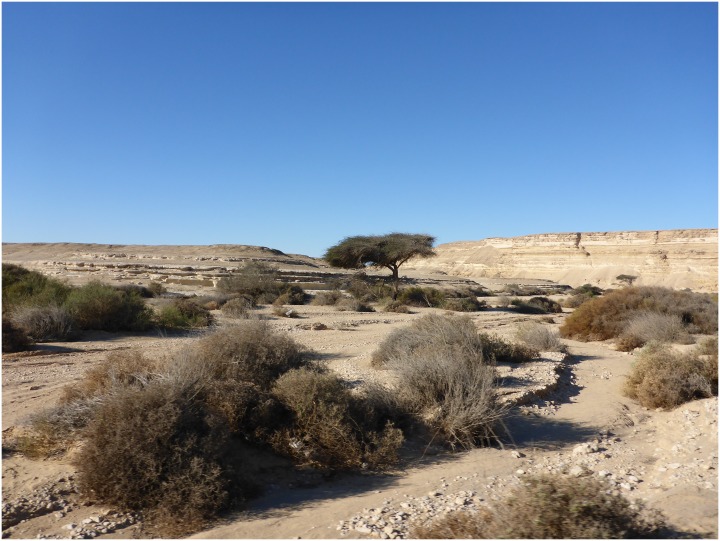
View of Wadi el-Sheikh. An example of the landscape and vegetation in spring (Photo E.C. Köhler).

The area is very arid, especially across the wadi terraces and plateaus. Vegetation can only be observed in the form of patches of desert shrubs and occasional tamarix and acacia trees along the Wadi's bed (Fig 2). However, erosion patterns suggest that the area has been subject to sporadic heavy rainfalls which frequently occur during the winter season in Egypt. Beside insects, reptiles, rodents, foxes and small birds, medium-sized desert fowl were also noted. Significantly, the area does not seem to have any active water sources today which makes it necessary to bring drinking water from the Nile Valley. It is probable that the area was less arid in prehistoric times and that the Wadi was a more or less active side stream of the Nile Valley, but this most probably did not apply after the aridization of north-eastern Africa had progressively increased from the early 3rd Millennium B.C.E. onwards. The climatic and ecological conditions in which the ancient mining activities took place are key research questions of this project as they have a direct impact on the logistics of mining and related activities.

The areas of the Wadi where chert was exploited cover a very large expanse of terrain where different modes and scales of exploitation can be studied in almost pristine conditions. Due to the remoteness of the area there has been—at least up until recently—very little human impact on the archaeological preservation for thousands of years. In some localities, the knapping areas which are exposed on the surface are barely covered by sand or secondary sediments. It sometimes looks as if the workers have just gotten up and left. There is very limited evidence for ancient cultural or natural transformation processes which may have negatively impacted the preservation of the site and there must have been a steady equilibrium between sedimentation of wind-blown or water-borne sand and erosion. Furthermore, the hardness of the chert has enabled the remains to sustain the elements of sun, wind and occasional rains very well. Only the surface material exhibits evidence of patina and limited thermoclastic cracking. The patina demonstrates the stability of the surface and lack of horizontal movement of the artifacts. Most surface artifacts have developed a dark patina only on the exposed surfaces, while the buried or face-down portions were much lighter and relatively unchanged. In other areas, especially low-lying ones, there has been a degree of sedimentation as many of the shafts and pits have filled up with windblown or washed-in sand over time, which however, has a favorable effect on the preservation of softer organic materials.

Overall, the chert mines at the Wadi are in a near perfect state of preservation, at least in those areas that are still difficult to access today. On the other hand, the past 10 years have seen a disturbing increase in modern cultural transformation processes. The construction of the Military Road has cut a wide path through the Wadi's terraces destroying large portions of major archaeological sites, and construction materials like sand and gravel were obtained locally, disturbing the surface for hundreds of meters out from the edges of the road. There are traces of heavy machinery having been engaged in moving the materials to the construction site which has already destroyed archaeological data. Furthermore, starting in 2015, a new high-voltage electricity power line was laid in the Eastern Desert connecting northern Egypt with the hydroelectric plants of the Aswan High Dam. The pylons for this power line were placed east of the Military Road and involved the construction of large concrete pedestals along the way. Our mission was able to record a number of localities already where these pylons destroyed chert deposits and mining sites, such as in L21 and south of L7. Furthermore, where there is construction going on there are people moving about and taking an interest in their surroundings. In a number of localities, especially near the roads and the electricity pylons, our mission noted evidence for modern illicit excavations at the mining sites, presumably in search for treasure. While there is little danger in artifacts being stolen from the site because lithic artifacts do not meet local definitions of 'treasure', these unchecked illegal excavations do have an impact on the preservation and quality of the archaeological contexts.

And finally, we have observed significant evidence for land reclamation projects at the bottom of the Wadi. For example, vast areas of the Wadi's mouth near the Nile Valley have already been claimed by settled bedouins for agriculture, and driving along its path toward the east showed traces of preparatory leveling of the Wadi bed with bulldozers. Traces of such activities have been observed some 5 km into the Wadi, i.e. just behind the first main loop and to the west of the Military Road. Our research thus takes place at a timely moment when much archaeological evidence can still be encountered in good quality contexts.

## Methods, aims and objectives of investigation

Three seasons of archaeological work have been conducted at the Wadi since 2014 by the University of Vienna Middle Egypt Project which is run in cooperation with the Ministry of Antiquities in Egypt, Deutsches Bergbau-Museum Bochum and the University of Virginia. The core project staff are the director (E.C. Köhler), three mining archaeologists (T. Stöllner, M. Klaunzer, F. Mustar), a lithics expert (E. Hart), a surveyor (A. Makovics) as well as accompanying local antiquities inspectors and support personnel. The number of archaeological specimens analyzed and included in this paper amounts to ca. 4900. These are today held in the mission's storehouse at el-Sheikh Fadl and are not accessible to the public. So far, the fieldwork has involved a combination of extensive and intensive surface surveys, geo-tagged photography, sampling and test excavations in order to arrive at a primary assessment of the terrain and archaeological evidence. In the course of this work 24 localities have been identified where mining activities can be observed, and many more are evident on satellite images. Most of these, i.e. at least 19 out of 24 localities, aimed at the extraction of chert. Test excavations and more intensive sampling and analysis of chert material were conducted in L1 and especially L20. The project aims to continue prospection, surveying and focused excavations in the future in order to fully comprehend the chronology, the mining and artifact technologies, expedition infrastructure and logistics, artifact distribution patterns and the motivation behind the long use life of this mining area.

The reasoning behind this approach is to contextualize the acquisition of chert and production of lithic tools on the background of ancient state economies, political economy, and specialized production based on modern theoretical approaches within Middle-Range-Theory and archaeological political economy [8, 9, 10]. These offer useful avenues of socio-economic contextualization of craft production, acquisition of raw materials, human resources as well as the financing of mining activities and the subsequent distribution of the products. For example, we hypothesize that the mining practices increase in complexity in step with the social and political transformations of life in the Nile Valley. Presumably during the early prehistoric periods the mining expeditions were of informal character and conducted by small groups of workmen with little internal organization or hierarchy since this is the case for the socio-economic organization of societies in the Nile Valley. Although socio-cultural and technological change can have many causes, we hypothesize that when the size and socio-economic complexity of societies increased during the Predynastic period, so did the demand for certain products including chert implements. This increased demand likely affected an increase of complexity in the organization of labor and resources [11] and probably also made necessary the control over resources and their exploitation, an area of research that is well-covered in the literature on archaeological political economy. We intend to investigate whether there is any evidence for state involvement at those localities that date to the Pharaonic period. State involvement could be indicated directly by inscriptional material, such as seals, clay sealings, papyri, graffiti or formal hieroglyphic inscriptions. Additionally, infrastructural evidence can indicate state involvement if the data points to large groups of workers provisioned from the Nile Valley. Comparison of the infrastructure organization and operation of the Wadi el-Sheikh mining activities to other known state-run Pharaonic expeditions could also provide evidence for state involvement. The question of state involvement in chert acquisition and lithic production is significant because the majority of known chert mines in world archaeology were exploited in prehistoric periods, i.e. by non-state societies, with few examples of chert exploitation in state societies [12, 13], even fewer of which had chert mining actually organized by the state. In state societies, which exerted state control over the production of tools, formal state-organized mining expeditions more commonly focused on other materials (e.g. obsidian, metals, stones for construction). The Wadi el-Sheikh is potentially one of the few known examples of state level involvement in the specialized acquisition and production of chert tools.

## Methods of chert mining

A sizable number of distinct mining areas have been recorded which document that a variety of methods were involved and that indeed no effort was spared by the ancient workers in order to extract the desired materials. It seems obvious that the miners were aware of the better quality of buried chert that was not exposed to the sun and extreme temperature differences which cause fissures and decay; thus 'mountain-fresh' chert was the preferred material. For example, even where there is evidence for the use of surface chert, it is accompanied by shallow small pits that reached non-exposed deposits. But what is far more common is that the raw material was excavated via sizeable extraction pits in areas of high quality chert deposits.

**Fig 3:**
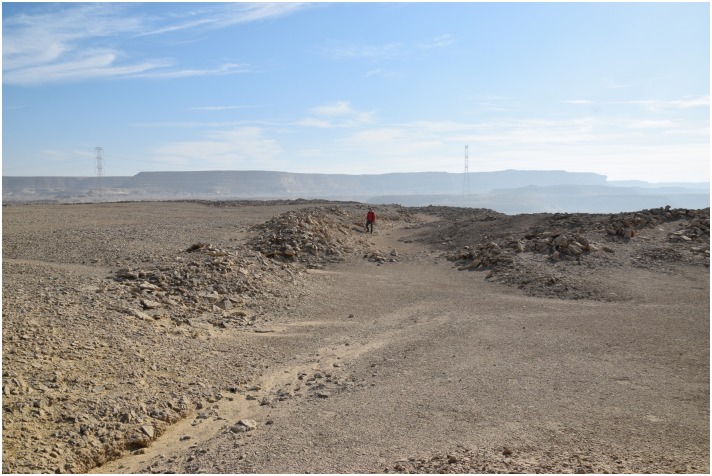
A mining trench in L20B. An opencast mine in form of a trench can be seen. On both sides of the trench heaps of stowing are piled up. In the background are the 'Military Road' and the new electricity pylons (Photo M. Klaunzer).

**Fig 4:**
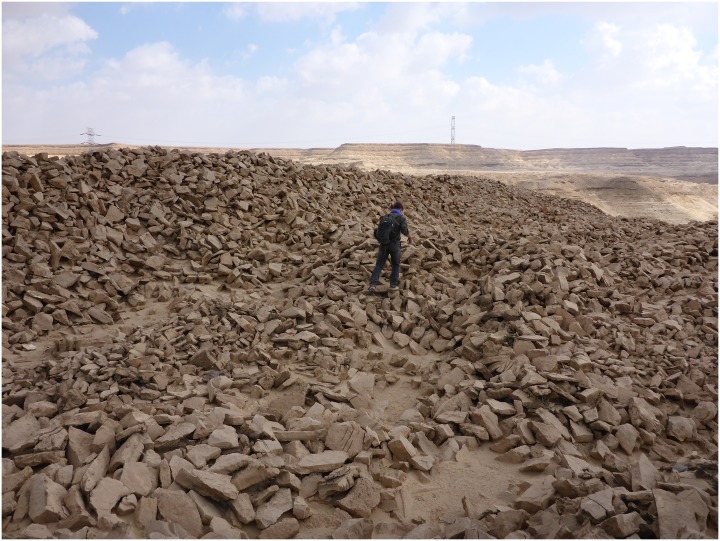
Spoil heaps in L20C. F. Mustar while climbing over ancient spoil heaps in L20C (Photo E.C. Köhler).

In other areas, for example where the natural deposits were easily visible and exploitable on the edge of a terrace, wide trenches of up to 3 m depth were cut from the side of the Wadi into the terraces in order to access the raw material (Fig 3). This, however, made necessary the removal of unwanted material such as the limestone in which the chert was embedded, which resulted in the creation of sometimes gigantic spoil heaps that completely altered the appearance of the ancient landscape (Fig 4).

**Fig 5:**
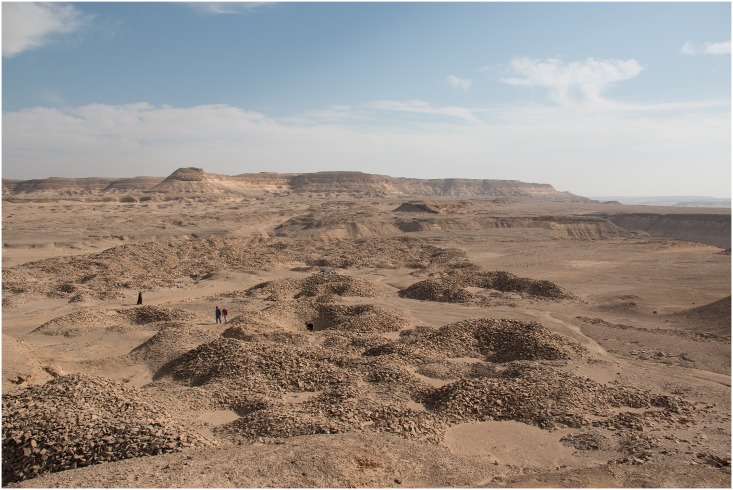
View of L5 looking east. The whole area up to the escarpment in the background is pockmarked with pits, which are now silted-up shafts, surrounded by circular or crescent-shaped spoil heaps (Photo T. Stöllner).

**Fig 6:**
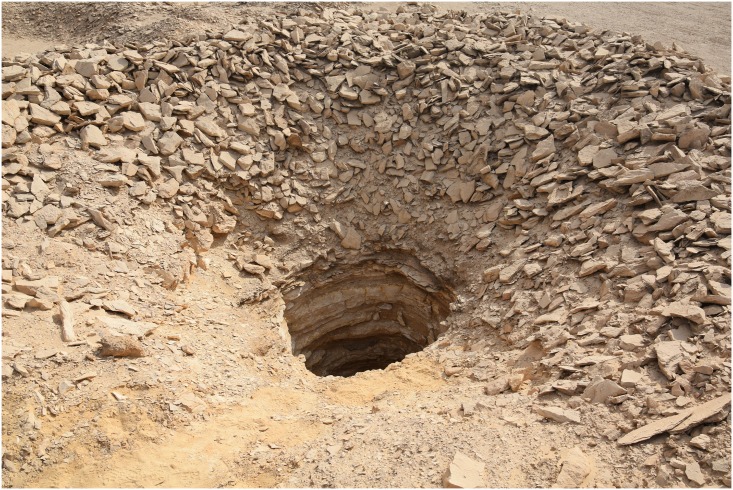
One of the vertical mining shafts in L5. The shaft descends vertically for about 5 meters and is surrounded by ancient excavation spoil (Photo M. Klaunzer).

Furthermore, underground mining also took place on the higher terraces where the chert deposits were buried below thick layers of limestone. Vertical shafts with a diameter of about 1–2 m and a depth of approx. 4–6 m were driven into the bedrock in order to exploit the deeper layers of good quality chert (Figs 5 and 6). The stowing of excavation spoil was first deposited in large heaps surrounding the shafts; as soon as the miners started to work horizontally underground, much of the stowing was then also left in abandoned horizontal exploitation chambers as the mining galleries drifted along following the chert deposits and thereby created complex underground cave systems. For example in L11, an area of almost 130 000 m^2^, at least 40 such shafts are easily discernible in satellite images. Furthermore, our mission was also able to document a large horizontal underground exploitation chamber in L20 of up to 150 m^2^ in area where high quality chert nodules were extracted (see below).

**Fig 7:**
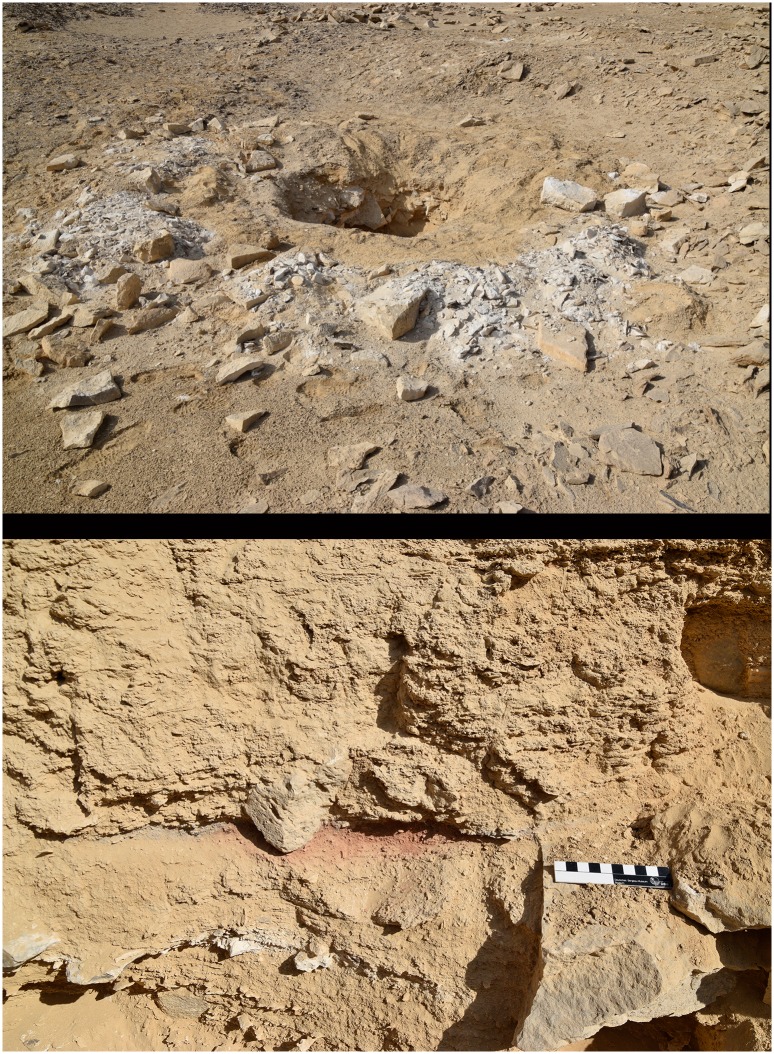
A modern looters' hole in L20C. The hole is surrounded by freshly excavated (white) debris including chert tools and debitage, Old Kingdom potsherds and a metal object. The image below shows a fireplace in the section of the looters’ hole (Photos M. Klaunzer).

**Fig 8:**
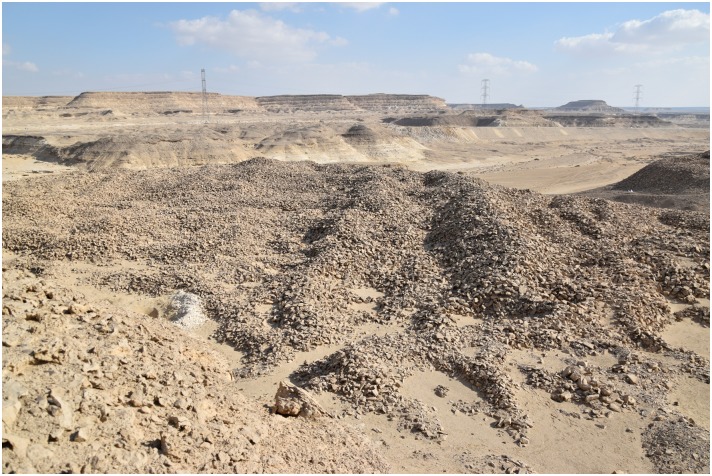
Spoil heaps in L20C. Pits, trenches and spoil heaps in L20C; on the left is a modern looters' hole with fresh (white) excavation spoil; the 'Military Road' and electricity pylons are visible in the background (Photo M. Klaunzer).

**Fig 9:**
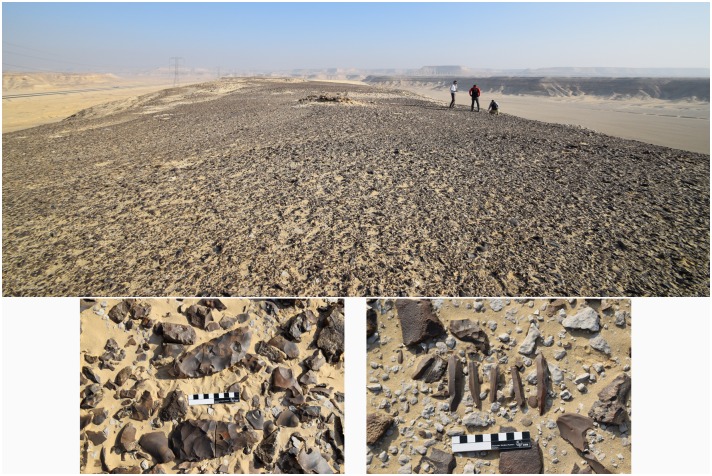
Chert at L19. There is a dense carpet of chert raw material, debitage and tools spread across this elongated ridge of L19 seen from the south. Below are detail views of the surface at L19 showing artifacts including bifacial preforms and blades (Photos M. Klaunzer).

Apart from the geological stratification of mineral deposits, there is also evidence for archaeological stratification suggesting that mining activities took place repeatedly or continuously in one spot over a long period of time. For example, in a modern looters hole (Fig 7) near the road in L20C we observed different stratified layers totaling more than 1 m thickness comprised of silty and water-borne sediments, large chunks of limestone debris, dense chert debitage as well as evidence for a fire place clearly embedded between the layers. This would suggest that the area around this hole was utilized by ancient miners for a considerable period of time. The debris that resulted from the mining and processing of chert can be extensive (Fig 8). The surface is often covered by a dense, ankle-deep carpet of raw material, clusters of knapping areas with half finished tools, cores and debitage spread over many thousand square meters (Fig 9). For example, some of our test excavations in four 1x1 m squares (= in total 4 m^2^) in L20 removed only about 2 cm of the surface material and yielded a sample of 4861 pieces of chert larger than 1 cm (see below). Many such deposits are likely stratified, as is visible in the nearby looters' holes. If the surface density is any indication, these deposits could yield over 60,000 lithic artifacts *per cubic meter* and even more including the pieces below 1 cm in size.

**Fig 10:**
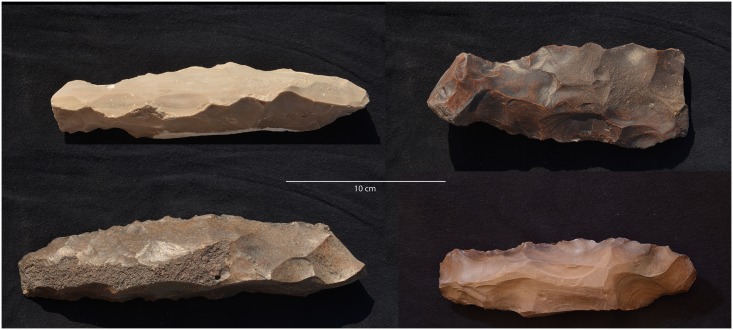
Examples of mining tools from Wadi el-Sheikh. Mining tools (picks) made of chert and found in mining sites dated to the early Pharaonic period based on finds of pottery and other characteristic artifacts such as bangles. (Photos F. Stangelberger).

The actual ancient excavation for the raw material was done by hand with the assistance of axes, rounded hammer stones, picks of chert and other materials such as quartzite, silicified limestone and possibly copper (see below). As surface finds and artifacts from our excavations have shown (Fig 10), the chert picks tend to have a handle-like narrowing or tapering end and are often of a relatively standard size with ca. 15–25 cm length, although pieces of a length of up to 50 cm are also reported [14].

## Chronology of the mining activities

The quarrying activities at Wadi el-Sheikh covered a very long span of time. Previous research has identified the presence of Predynastic and Pharaonic remains in the Wadi, and our latest research has provided primary evidence unambiguously confirming the presence of many Pharaonic mining areas throughout the Wadi and extending the chronological span of human activities to earlier time periods.

**Fig 11:**
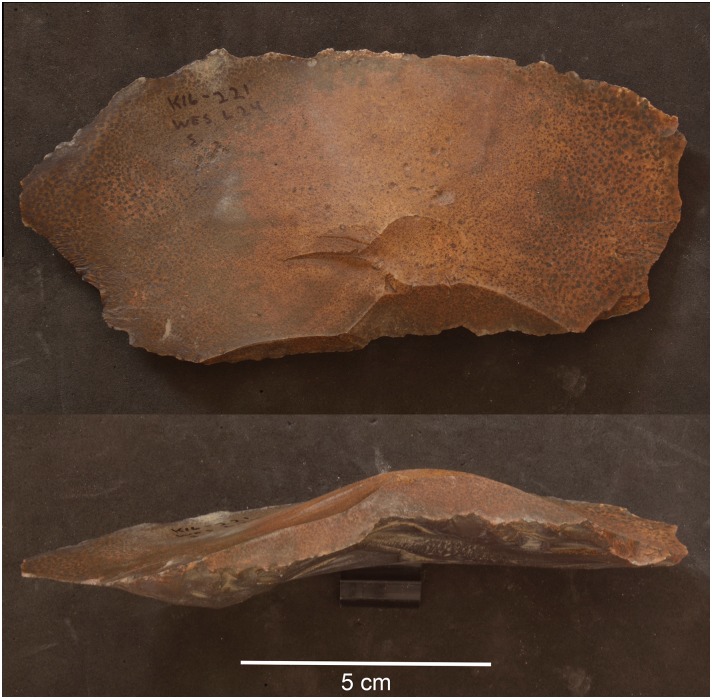
Example of a side-blow flake from L24. This scraper was made on a flake using side-blow like reduction technology. Side-blow flake tools can be considered typical of the Neolithic period in Egypt. (Photos F. Stangelberger).

**Fig 12:**
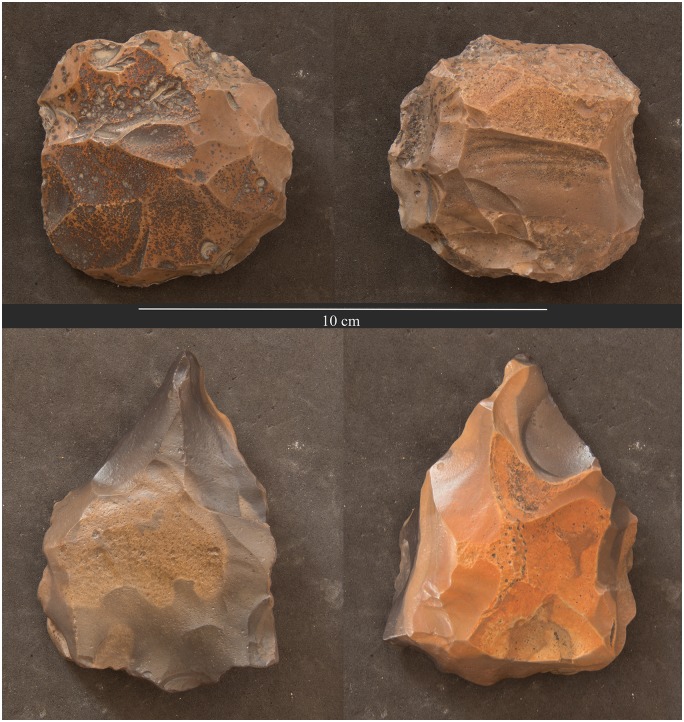
Example of Levallois cores from L20. Two examples of Levallois cores found on the surface of L20 with heavy weathering and patina. (Photos F. Stangelberger).

For example, our mission was able to locate pottery in association with the mining works which often assisted in narrowing down the relative date of ancient and modern activity. Although chronologically diagnostic pottery has not yet been found at all mining areas, more than half of surveyed sites can be dated to the Pharaonic and earlier periods on the basis of the pottery and other artifacts. For example, a survey at L24 has yielded material entirely different from the Pharaonic and modern sites and most likely dates to the Neolithic or Chalcolithic period. While none of the artifacts found there to date are perfect chronological markers, the artifacts and the mining method differ from that of other sites. The blades from L24 are much larger and technologically different than the blades found at Pharaonic sites. Other debitage and tools derive from a technology reminiscent of side-blow flake reduction technology (Fig 11). Side-blow flakes are characteristic of Egyptian Neolithic sites of the 5th Millennium B.C.E. [15, 16] and have sometimes been found in Chalcolithic sites [17]. Interestingly, this locality is also one of the most remote of mining activities that this mission has surveyed to date (Fig 1). Additionally we have identified Middle Palaeolithic stone tools in a number of surface contexts throughout the Wadi, including Levallois cores (Fig 12). The Middle Paleolithic artifacts have heavy patina and weathering on all sides, differentiating them clearly from the significantly younger lithics. So far we have not identified chert extraction that can definitively be associated with the Palaeolithic materials, but considering that Middle and Upper Palaeolithic chert extraction has been identified in other parts of Egypt (Table 1), we will explore this possibility in future field seasons.

**Table 1:** Chronological table.

Ca. years B.C.E.	Relative chronological / historical period	Chert mines at the Wadi el-Sheikh	Other chert/flint mines in Egypt
1550–1070	New Kingdom	X	Tell el-Amarna, Umm Nikhaybar, Hierakonpolis
2050–1550	Middle Kingdom—Second Intermediate Period	X	
2600–2050	Old Kingdom—First Intermediate Period	X	Galala North/Sannur
3300–2600	Naqada III / Proto- and Early Dynastic	X	Galala North/Sannur
4000–3300	Naqada I+II / Chalcolithic (Predynastic)	X	Hierakonpolis, Ain Barda
5100–4000	Neolithic	X	
24 000–7000	Late Palaeolithic / Epipalaeolithic		
50 000–24 000	Upper Palaeolithic		Nazlet Khater-4
175 000–70 000	Middle Palaeolithic	?	Taramsa Hill

While the lithic artifacts in the Wadi have so far allowed us to identify broad chronological trends, the nature of the activities poses unique challenges for making fine chronological distinctions within the Pharaonic period, and for narrowing down the dates of different Pharaonic mining localities relative to one another for a number of reasons. The vast majority of the remains left behind by workers in the Wadi consist of cores, debitage, rough-outs, preforms or semi-finished tools whose manufacturing process was not completed due to breakage. The intended products, finished tools or blanks for tools must have been transported to the Nile Valley. Conversely, research on Pharaonic stone tools has up to this point focused mainly on typologies of finished tools—with less work focused on understanding the cores and debitage that resulted from producing those tools. Hence, what was left behind at the Wadi has few equivalents in the Nile Valley where the finished products were used in daily life, ritual or funerary contexts. The artifacts in the Wadi therefore do not find many close parallels in the archaeological record of the Nile Valley which could be used for refined typological dating [14]. Furthermore, the study of Pharaonic stone tools is itself a small field to date, where most of the publications are site reports, and there are only a few publications drawing together many studies to reach broad conclusions [18, 19]. Therefore this project aims to take advantage of the unique opportunities offered in Wadi el-Sheikh to study Pharaonic stone tool production remains, correlate them to known and dated tool types, and gain a better understanding of production sequences and their change over time, along with more precise dating of the Pharaonic activities in the Wadi.

**Fig 13:**
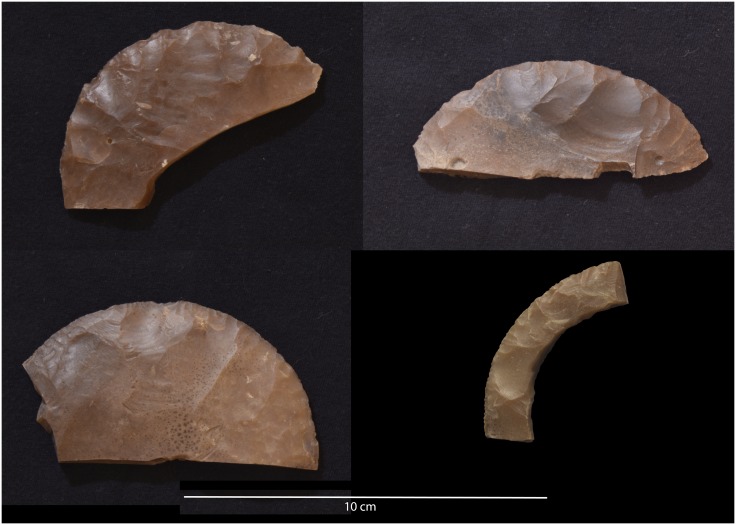
Examples of bangle preforms and a fragment from L20. The bangles were produced from bifacially retouched discs where the center was then removed creating a trifacially retouched ring that was later often polished. The pieces shown here were discarded due to breakage in the manufacturing process (Photos F. Stangelberger).

**Fig 14:**
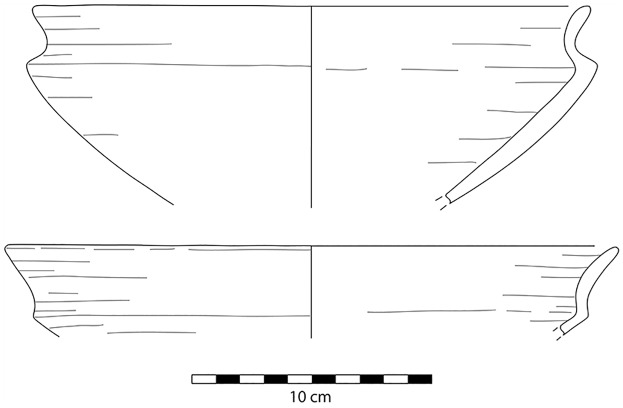
Old Kingdom pottery found at L20B. Line drawings of two examples of Meydum bowls from L20B. Bowls of this kind are classified as Meydum Ware of the Old Kingdom (Drawings E.C. Köhler).

Fortunately, for the purposes of chronological dating, there is one very exceptional group of artifacts whose preforms and half-products are quite distinct and chronologically diagnostic. These are chert bangles which in the Nile Valley can be dated to the Naqada III or Proto-and Early Dynastic period [18, 20, 21, 22, 23, 24, 25]. In L20 we found significant evidence for the manufacture of such bangles (Fig 13), at least up to the point where the trifacially retouched preforms had been achieved. In most cases where such bangles were found in the Nile Valley, though, they were very delicate and polished pieces of jewelry, only in rare instances the coarser, unpolished varieties are known [20]. Our mission was also able to locate Pharaonic pottery in association with the mining works of Locality 20 (Fig 14), for example fragments of the so-called Meydum Ware, a highly diagnostic ceramic ware of the Old Kingdom. This is an important observation because the bangles, the Old Kingdom pottery as well as the evidence for archaeological stratification in the same area suggest that mining and chert processing activities indeed took place in this spot over a long period of time (see below).

There are a few other instances where archaeologists were able to draw a direct comparison with well-dated Nile Valley material. For example Pawlik was able to identify material in the nearby Pharaonic settlement at Kom el-Ahmar/Sharuna that seems to have been mined at the Wadi. This evidence indicates that chert exploitation took place at least during the Early Dynastic, Old and probably Middle Kingdom Periods, i.e. over much of the 3rd and early 2nd Millennium B.C.E. [20]. Also, Tillmann observed a close relationship between the raw material of lithic artifacts found in Middle and New Kingdom layers at Tell el-Dab'a in the eastern Nile Delta and the chert material from Wadi el-Sheikh, i.e. at a distance of 300 km [26, 27]. Furthermore, Harrell has recently [28] dated the mining activities at the Wadi to an even broader time period between the Predynastic and New Kingdom, thus covering at least three millennia from the early 4th until the late 2nd Millennium B.C.E. (Table 1). Our new work was able to further extend the beginning of activities to probably as far back as the 5th Millennium B.C.E., and possibly to the Middle Palaeolithic period.

Wadi el-Sheikh has the longest time depth of chert exploitation of any mining site in Egypt dating at least from the Neolithic until the New Kingdom and covering thousands of years of important socio-economic and political transformations in the Nile Valley. We will demonstrate the significance of the Wadi for these aspects with the example of L20, a locality where we have gathered the most insight and definition up until this point.

## Chert mining at L20

L20 is a plateau located on the northern side of the Wadi just to the west of the Military road (Fig 1) and extends over 1.6 km from north to south, thereby covering more than 300 000 m^2^. On the basis of ceramic and lithic finds, L20 dates from the Early Dynastic through Old Kingdom periods, i.e. approximately 3100–2200 B.C.E. The locality had clear boundaries based on the presence and concentration of mining features and artifacts. We arbitrarily divided the locality into three sub-localities (L20A, B and C) based on the presence of natural features to make this large area more manageable. The whole area is characterized by enormous spoil heaps, opencast mines, trenches, pits, underground galleries and possibly shafts, numerous lithic knapping areas and artificially built stone structures which most probably served as shelters for the miners.

**Fig 15:**
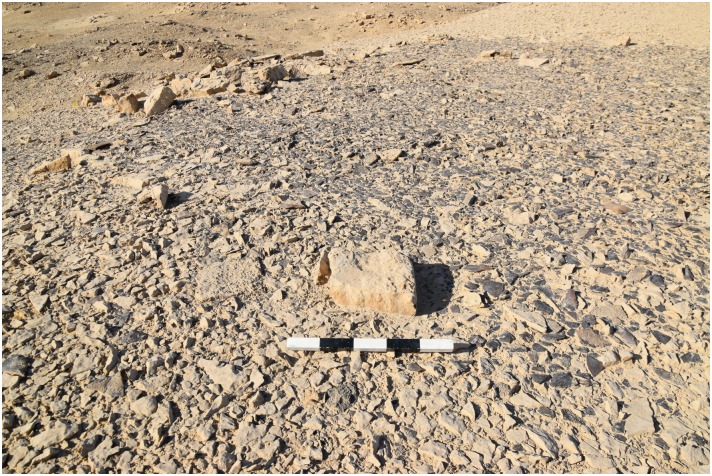
Knapping area with a central stone at L20B. There is a wide circle of chert debitage and tools surrounding a central flat stone, which we interpret as a seat for the tool knapper (Photo M. Klaunzer).

**Fig 16:**
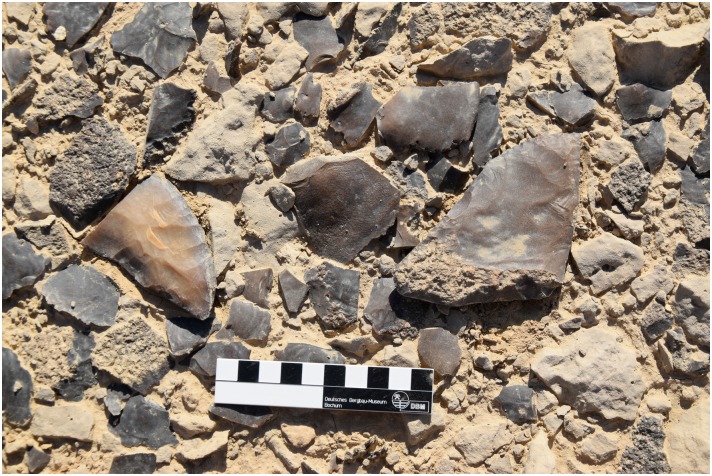
Examples of tools produced in L20B. Bifacial knife fragments and flakes from tool production (Photo M. Klaunzer).

**Fig 17:**
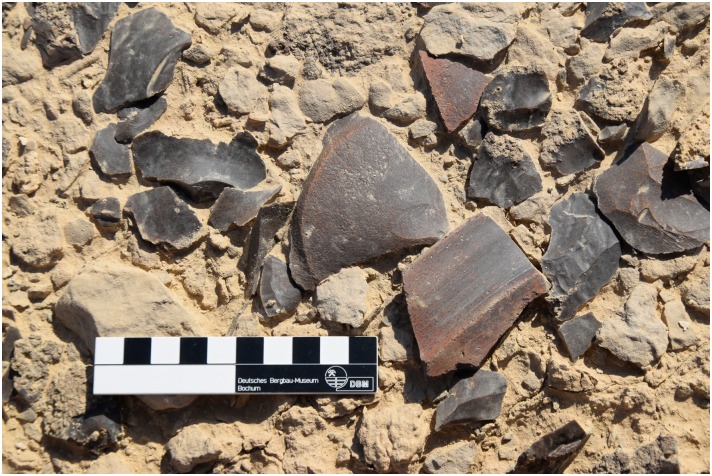
Old Kingdom pottery fragments in situ at L20B. One of the fragments of a Meydum bowl as shown in Fig 14 associated in situ with chert debitage (Photo M. Klaunzer).

For example at L20B extensive opencast mining in the form of trenches were made near the edge of the plateau. The related spoil heaps are piled up on both sides of the trenches and especially near the edge (Fig 3). A number of smaller stone structures were observed, which may be wind shelters or small houses. Chert picks, hammer stones and other mining tools were used to extract the raw material. The exploited chert was then processed in knapping areas directly next to the opencast mines. Several knapping sites in L20B had central stones, which may be interpreted as seats for the knappers (Fig 15) surrounded by a wide circle of dense debitage (flakes, semi-finished products, debris etc.). Different kinds of tools were produced here such as blades, knives and other bifacial tools (Fig 16). A number of ceramic fragments permit this particular locality to be dated to the Old Kingdom (Fig 17).

**Fig 18:**
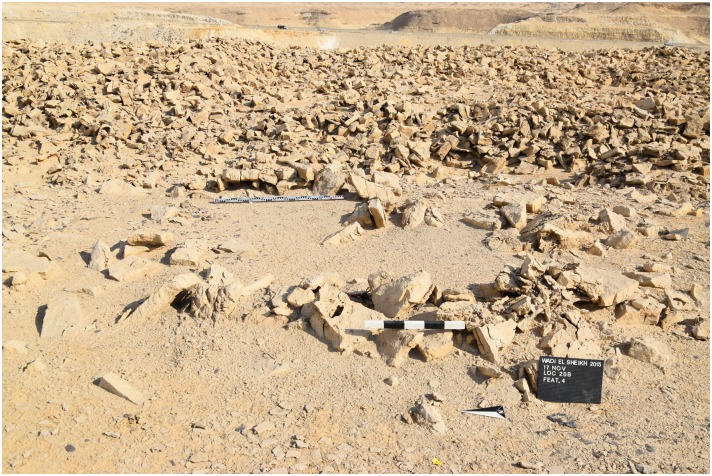
Stone structures at L20B. The image shows Feature 4, a possible house structure, in Area 1 L20B, in the background are mining spoil heaps and debris (Photo M. Klaunzer).

**Fig 19:**
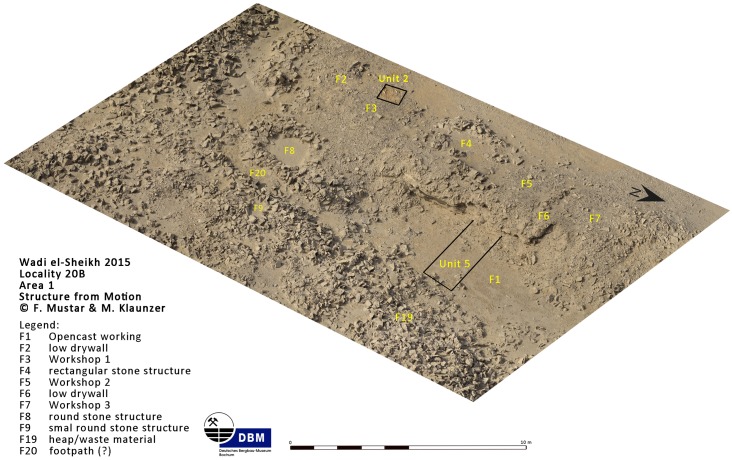
Structure from Motion-enhanced photograph of Area 1 in L20B. The image shows Units 2 and 5 as well as numerous features recorded in Area 1 of L20B (Image F. Mustar and M. Klaunzer).

**Fig 20:**
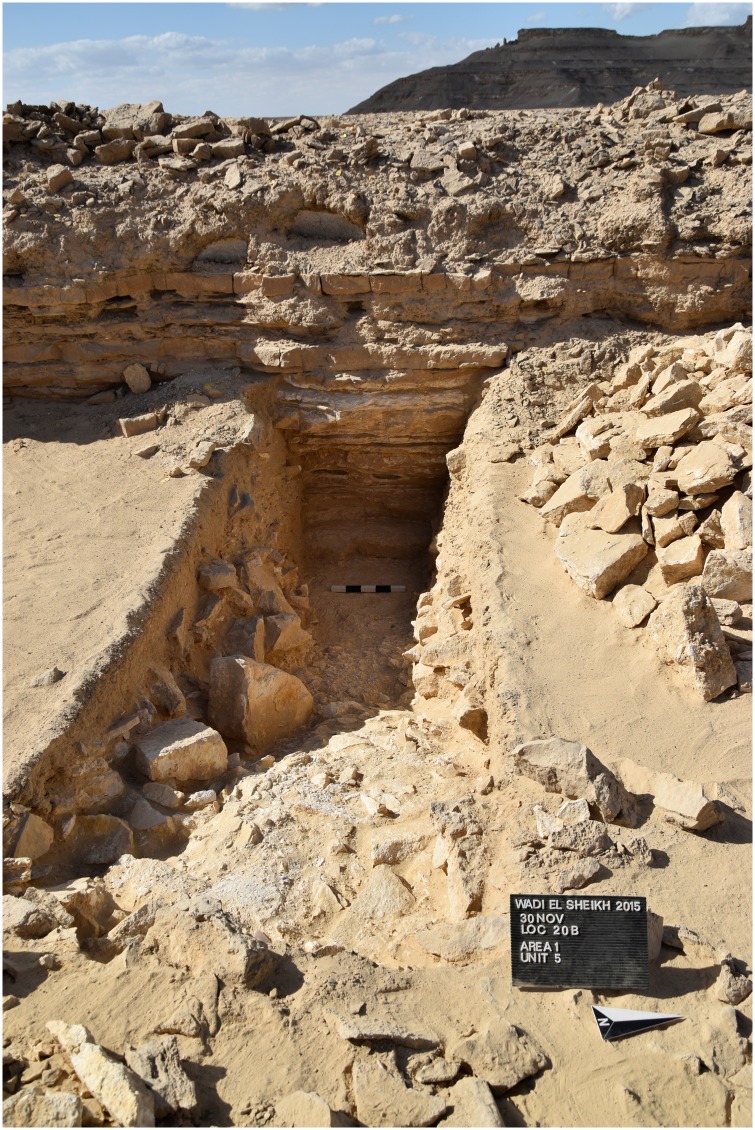
Unit 5 in L20B after excavation. The image shows Unit 5 and the section of the mining trench from the east (Photo M. Klaunzer).

**Fig 21:**
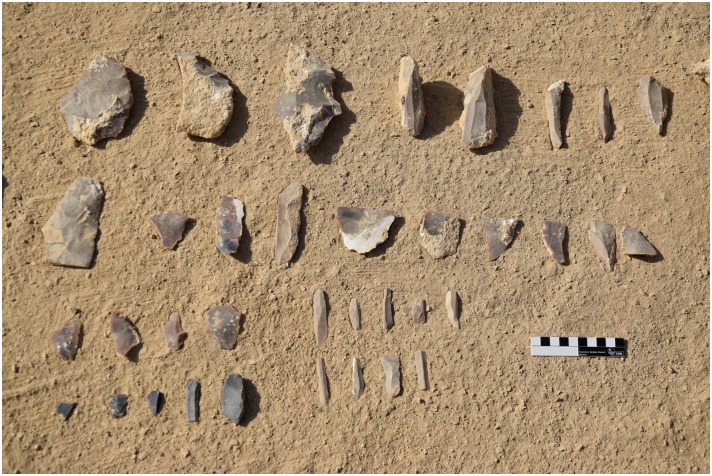
Artifacts including cortical flakes, cores and blade fragments from Unit 5 in L20B. (Photo M. Klaunzer).

The open ditch extraction can sometimes be traced over hundreds of meters along the edge of the plateau. It seems as if the whole plateau was excavated from the edge inwards. The advantage of this method is that once the layer of preferred raw material was identified at the edge it could easily be followed and the opencast mining continued for as long as necessary. Near these opencast mines on top of the plateau, several knapping sites, partition walls, possible house structures (Fig 18) in the form of low stone dry walls, and other stone structures can be observed. The opencast mines are today silted up. In order to understand how deep the ancient miners had dug to exploit the raw material an excavation unit of 4 x 1.5 m area (Unit 5, Figs 19 and 20) was placed across a large trench-shaped open ditch thereby cutting through layers of secondary fill and exposing the stratigraphy. The results of our excavations suggest that the ancient miners had dug a trench of about 3 m depth into the natural landscape and broke through different layers of limestone and other rocks. Here, the exploited raw material was tabular and flat nodular chert which is still visible as thin horizontal bands in the section of this trench. The archaeological layer directly upon the bedrock contained pieces of raw material, debitage as well as unfinished artifacts, mostly with cortex remaining (Fig 21). Additionally, broken mining tools (fragments of axes, hammer stones etc.) for exploiting the raw material were also found, one of them directly upon the natural bedrock.

**Fig 22:**
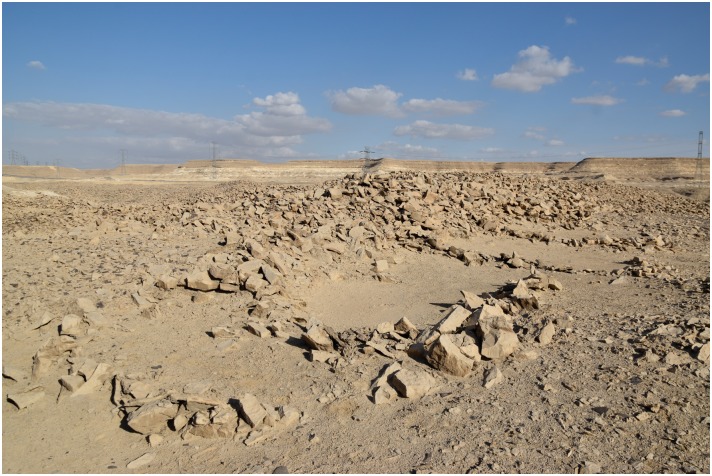
Building structure (Feature 24) at L20C. The building shown here comprises four to five adjacent chambers of comparable design, all oriented in the same direction and with the entrances in equivalent locations. (Photo M. Klaunzer).

L20C is characterized by even more extensive exploitation and production activities. There are enormous spoil heaps, dozens of workshops and various stone features. The features also include the remains of large-sized buildings with multiple rooms (Fig 22).

**Fig 23:**
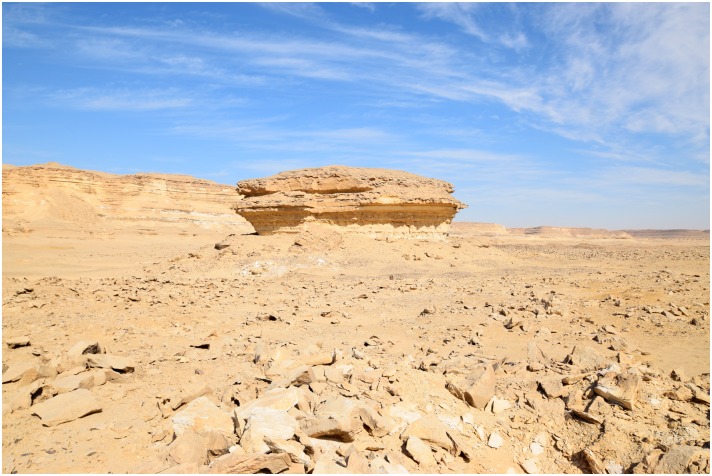
The landmark rock formation in L20C. The rock formation is surrounded by chert knapping areas on all sides (Photo M. Klaunzer).

There is a single tall rock formation that represents a useful landmark in this area (Fig 23). This rock formation was probably used as a shelter in ancient times, where the workers found protection from the sun and wind, and built fireplaces, such as one hearth which was observed in the stratigraphic profile visible in a looters' hole next to this rock formation. Notably, there are more than a dozen concentrated knapping areas all around this landmark that demonstrate its importance in the production of artifacts at the Wadi. The whole area to the north, east and south of this rock is covered with evidence of immense workings to exploit raw material from deep layers. It is in this area that relatively deep archaeological stratification was observed.

**Fig 24:**
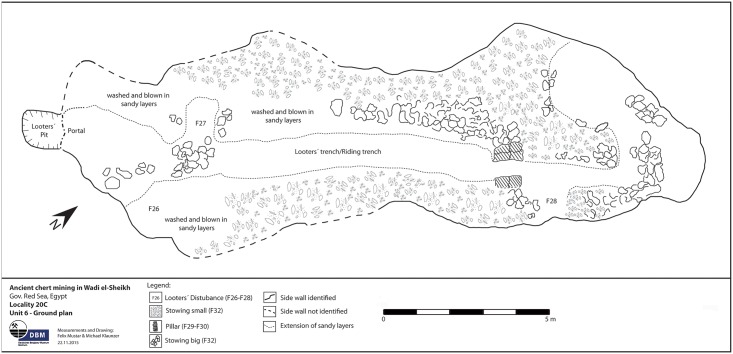
Plan of the exploitation chamber in L20C. (Image F. Mustar and M. Klaunzer).

**Fig 25:**
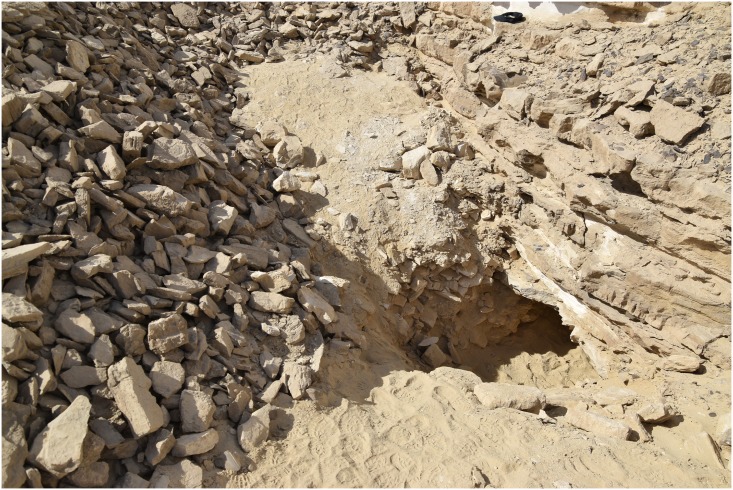
Entrance to the underground mining chamber in L20C. The chamber was started from an open-cast mining trench whose side is visible on the right. Modern looters excavated through debris and secondary fill of the trench and into the underground exploitation chamber (Photo M. Klaunzer).

**Fig 26:**
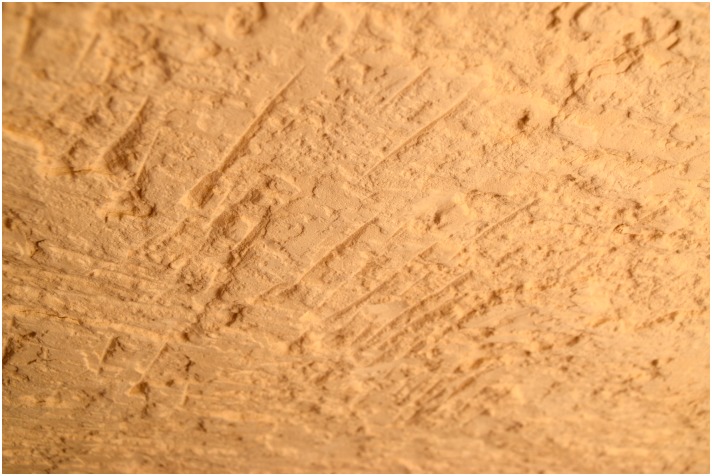
Pick marks on the ceiling of the exploitation chamber in L20C. The pick marks are regular and cover the entire ceiling (Photo M. Klaunzer).

**Fig 27:**
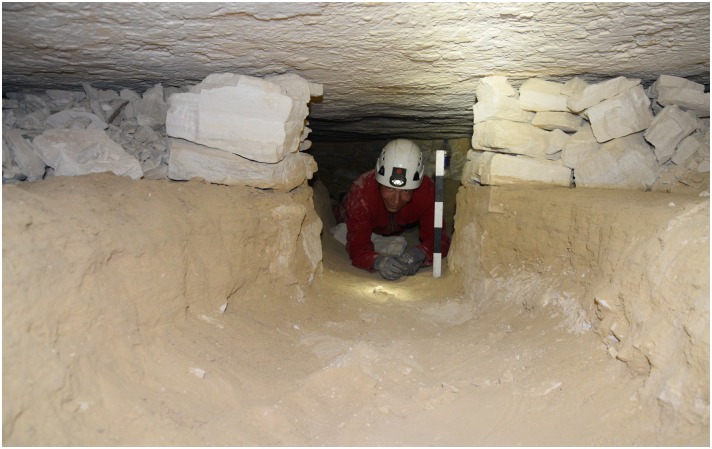
Inside the exploitation chamber in L20C. F. Mustar at the end of the chamber looking out. To the left and right are stone pillars and stowing; in the foreground are layers of ancient sand fill through which the looters had dug a narrow trench (Photo M. Klaunzer).

**Fig 28:**
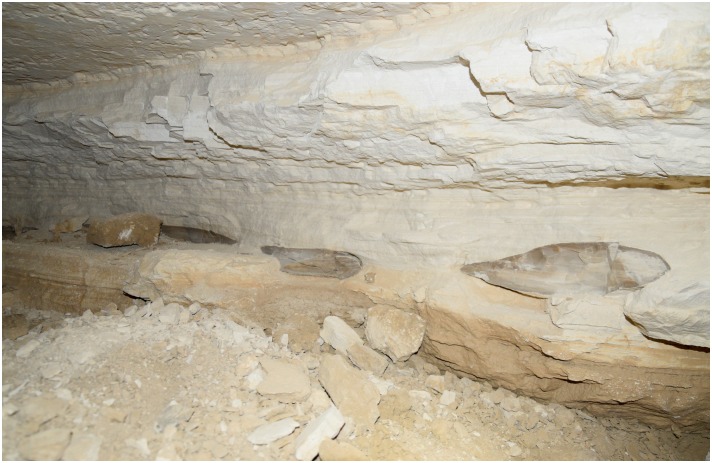
Raw material in the mining cavity at L20C. View of the back wall of the exploitation chamber with chert nodules in situ (Photo M. Klaunzer).

Numerous secondary pits were noted in L20 which we interpret as modern looters’ holes as the area is close to the Military Road, and the disturbances revealed many artifacts without patina and were sometimes accompanied by modern trash. Exploration of one of these holes in L20C lead us directly into an ancient horizontal exploitation chamber (Figs 24 and 25). Initially, the entrance to the underground gallery was started from an open ditch. The looters had dug out loose sand and thereby reopened an originally narrow area (approx. 0.6–0.7 m wide) that the ancient miners had left free of waste material. The chamber is approximately 19 m long and 7–8 m wide, the height is about 0.70–0.75 m. The ceiling of the whole chamber shows regular tool marks (Fig 26). The stowing was piled up on both sides and two possible retaining pillars were built by piles of limestone blocks (Fig 27). At the end of the chamber, a working cavity opens up where large pieces of nodular chert are visible still embedded in the bedrock (Fig 28). Some large stone axes used for the exploitation of raw material were found inside the mine, but were probably displaced by the modern looters. One of these mining tools shows a bright green discoloration on one side, possibly deriving from copper. This may be an indication that copper/bronze objects were also used for mining in the Wadi, although we have not located such tools yet.

**Fig 29:**
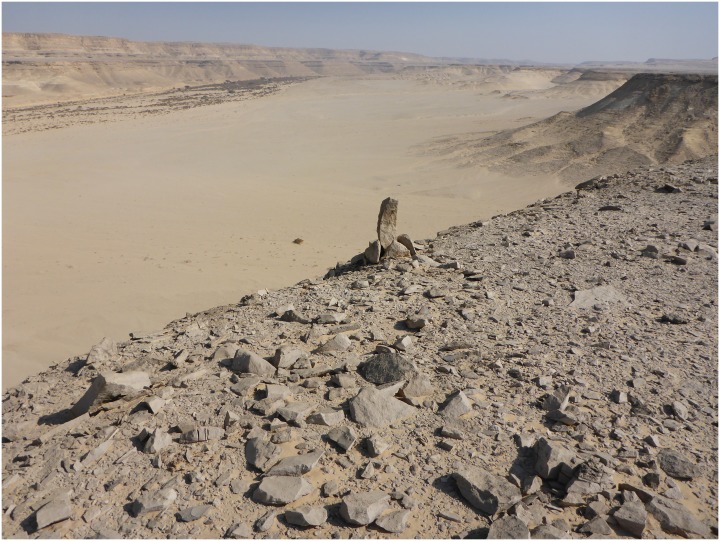
A stone cairn at the edge of the plateau near L6. This is one of several such stone markers placed along the edge of the plateau to mark the location of quarry sites in L6. They are well visible from the bottom of the Wadi (Photo E.C. Köhler).

**Fig 30:**
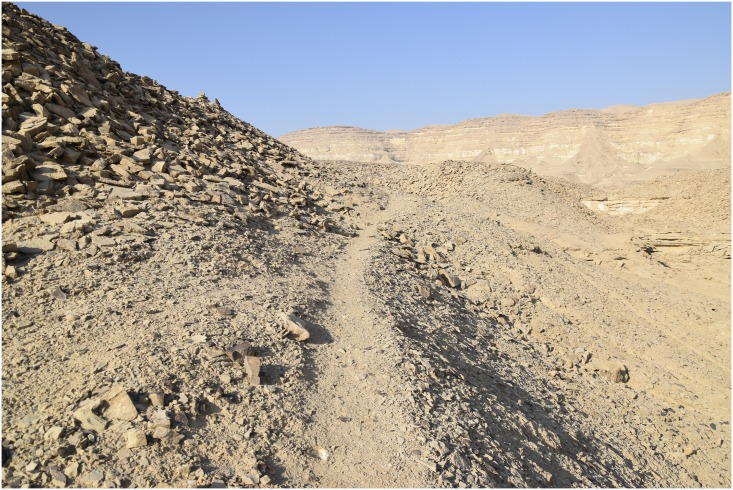
A trail and stone markers at L20C. A trail leads through massive mining spoil heaps and debris in L20C; two stone cairns can be seen half way along the left slope (Photo M. Klaunzer).

Along the edges of the plateaus there are often clearly visible stone markers or cairns set up in the form of vertical stone slabs or small piles of stones (Fig 29), sometimes combined with distinct trails leading from the Wadi bed up to the terraces and mines (Fig 30) [4]. The trails are too wide to be natural animal tracks, but to this day they provide easy access to the mining sites and since there are no other periods of intensive use evident besides the early Pharaonic mines in L20, the trails may have been used by the ancient workers and their transport animals laden with tools, water, food and, eventually, chert.

### Investigation into lithic production observed at L20

The three main lithic reduction technologies evident at L20 were for the production of prismatic blades, bifacial tools (knives), and bangles (e.g. Figs 10, 13, 16 and 21). The blades were used to make elements of sickles, which were an important kind of tool for the agricultural work that supported the Ancient Egyptian economy. Bifacial knives were probably used for many purposes; including the slaughter of cattle for offerings, as depicted in contemporaneous art scenes.

**Fig 31:**
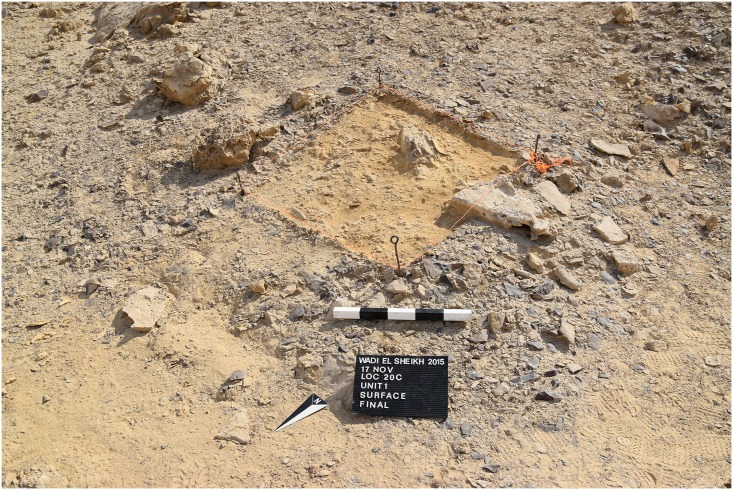
Unit 1 at L20C. Unit 1 after excavation of the top 2 cm of material; it produced more than 2100 pieces of chert which primarily resulted from bifacial tool production (Photo M. Klaunzer).

In order to understand how lithic production was organized at the site, we placed four 1x1 meter surface collection units in different areas across L20 where we collected lithic material from the surface to only 2 cm down (Fig 31). In some cases after the removal of the surface material, artifacts could be seen in the ground indicating that more cultural layers probably exist below the surface. In other cases there were no more artifacts apparent after removal of the surface material.

As we have already shown in the previous sections, the amount of chert extracted at L20 alone is enormous. Hence, the primary methodological issue for analyzing this material is how to deal with such large quantities of artifacts in a timely manner. The initial survey of L20 indicated that much information about the type or distribution of reduction technologies might be evident simply in the size of the artifacts. For instance during our surface surveys we noted that areas where the activities seemed to relate to raw material extraction and initial shaping the artifacts were often very large, whereas in other areas with concentrations of artifacts related to bifacial tool finishing, such as preforms and thinning flakes, the artifacts were noticeably smaller. In order to explore this, the artifacts were first separated into arbitrary size categories as follows: <1cm, 1–1.5cm, 1.5 -5cm, 5-10cm, 10-20cm, and 20+cm (pieces of a size of less than 1cm were collected but due to their very large number they have not been fully analyzed yet). The artifacts were then sorted into categories of tool, core, debitage, and debris, and specific data pertinent to each category were recorded. Metric data including maximum length, width and thickness were collected for all the cores, select tools, and the few complete blades. Additionally maximum width and thickness were measured for the blade fragments. Measurements were taken with metal digital calipers.

**Fig 32:**
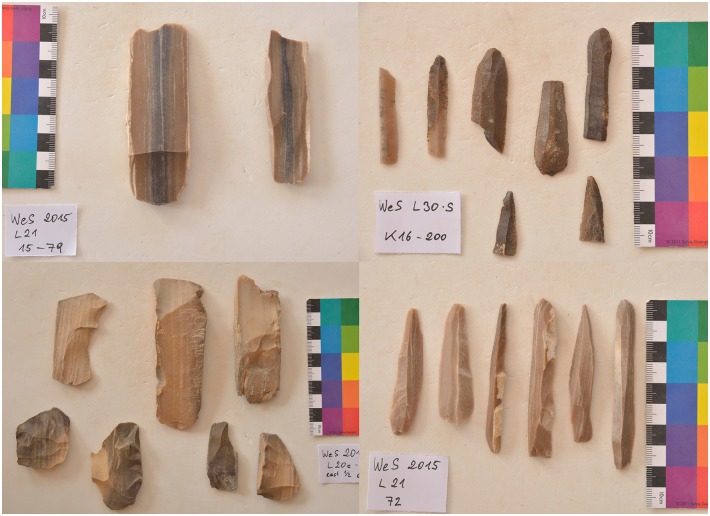
Examples of chert artifacts from Wadi el-Sheikh. The pieces shown here illustrate the variety of the raw material colors. Left: Core tablets off of tabular blade cores. Right: Blades. Note that the artifacts in the upper right have patina development. (Photos F. Stangelberger).

Artifacts at L20 were made from two main types of raw material: chert and a coarser material, probably silicified limestone, but chert was the most common material (see Table 2). However, these raw material designations were based on macroscopic observations only, and are accordingly provisional. Harrell suspects that much of the 'chert' in the Wadi may actually be more of a silicified limestone than an actual chert/flint. True material identification will have to await petrographic and chemical analysis of the proportions of quartz and calcite in future seasons. The main kind of chert found at L20 occurred as tabular pieces or nodules with cortex that ranged from rough and knobbly, to slightly rough and chalky, to very chalky. Raw material samples and freshly exposed artifacts from recent looter's holes showed that the main variety of chert processed into artifacts at the site can best be described as a light brownish gray, ranging from Munsell colors 10YR 7/2 light gray, to 10YR 6/3 pale brown, and to 10 YR 5/2 grayish brown, often with a dark brown to black center or with light banding (Figs 21 and 32). The artifacts and raw materials that were exposed on the surface, however, had developed a very dark patina (e.g. Figs 9 and 10 (top right), Figs 11, 12 and 17). Previous descriptions of Wadi el-Sheikh chert as dark brown or black may not have made a clear distinction between artifacts with and without patina. One important technology studied at L20 was the production of bangles. Pieces of bifacially retouched chert discs and a bangle fragment indicate that the production of this jewelry took place in L20C according to the following steps: a thin tabular piece of chert was bifacially prepared into a circular disk-like preform which was then hollowed out by means that may have included drilling, and pressure flaking to hollow out the central portion of the disk, and eventually create a complete trifacially retouched bangle (Fig 13).

**Table 2:** Counts and percentages of raw material types by unit (Units 1–4).

	Unit 1		Unit 2		Unit 3		Unit 4	
Raw Material	Count	%	Count	%	Count	%	Count	%
Non-chert	3	0.14	3	0.58	590	97.2	12	0.74
Chert	2110	99.86	516	99.42	17	2.8	1610	99.26
Total	2113	100	519	100	607	100	1622	100

#### Results of lithic analysis

A number of differences could be observed across the site in terms of raw materials, artifact density, artifact size and reduction technology. Artifacts in Units 1, 2, & 4 were almost entirely chert, with less than 1% of the artifacts made from other materials, usually silicified limestone (Table 2). Unit 3 however was exactly the reverse, over 97% of the material was silicified limestone, and only a few pieces were chert.

Table 3 shows the debitage categories for L20 Units 1–4. Major differences in the debitage categories of the units are evident, and show that the focus of production was different in each of the units. Units 2 and 3 both had a narrower range of types of debitage, and higher percentages of angular debris. Units 2 and 3 also had a much lower density of artifacts compared to the other two units. Units 1 and 4 each had three to four times as many artifacts as Units 2 and 3, across the same 1x1 m sized areas. These lower densities probably relate both to the kinds of reduction carried out in those areas, and to the frequency of activities. Units 1 and 4 are both stratified deposits, as more material could be seen continuing in the next layer after the surface removal. Units 2 and 3 however seemed to be confined mainly to the surface.

**Table 3:** Comparison of debitage categories between L20 Units 1–4. Debitage counts include complete and proximal pieces.

		Unit 1		Unit 2		Unit 3		Unit 4	
Category	Type	Count	%	Count	%	Count	%	Count	%
Tools	Tools	27	1.28	11	2.12	3	0.49	46	2.84
Cores & Core Trimming	Cores	14	0.66	7	1.35	2	0.33	45	2.77
	Core tablets	1	0.05	0	0	2	0.33	36	2.22
	Crested blades	0	0	0	0	0	0	7	0.43
Debitage	Flakes	265	12.54	92	17.73	205	33.77	238	14.67
	Prismatic blades	20	0.95	0	0	0	0	58	3.58
	Irregular Blades	10	0.47	1	0.19	0	0	38	2.34
	Plunging blades	0	0	0	0	0	0	2	0.12
	Thinning flakes	**258**	**12.21**	**33**	**6.36**	**23**	**3.79**	**96**	**5.92**
	Early stage thinning flakes	91	4.31	22	4.24	0	0	33	2.03
	Alternate flakes	61	2.89	18	3.47	3	0.49	24	1.48
	Burin spalls	2	0.09	0	0	0	0	0	0
	Other flake types & Ind.	243	11.5	27	5.2	47	7.74	133	8.2
Debris	Medial & distal flake fragments	468	22.15	127	24.47	170	28.01	417	25.71
	Medial & distal Trap. blade frags	51	2.41	1	0.19	5	0.82	96	5.92
	Medial & distal Irreg. blade frags	11	0.52	2	0.39	2	0.33	17	1.05
	Chips (1–1.5cm)	**431**	**20.4**	**44**	**8.48**	**38**	**6.26**	**122**	**7.52**
	Large tabular pieces	0	0	3	0.58	0	0	1	0.06
	Angular debris natural /artificial	91	4.31	96	18.5	90	14.83	78	4.81
	Thermal spalls & fractures	69	3.27	35	6.74	17	2.8	135	8.32
	TOTAL	2113	100.01	519	100.01	607	99.99	1622	99.99

The differences in production seen from the debitage categories are supported by the data for cores, tool classes, and artifact sizes. Unit 4 has by far the most evidence for blade production both in terms of quantity and kinds of cores. Over 80% of the cores are from the production of prismatic blades. Blade cores were also found in Unit 1, but not in Units 2 or 3. Initially tested nodules are the most common core type in Units 1, 2, and 3.

All of the units have a low percentage of tools overall (0.5-less than 3%) (Table 3). Units 1 and 4 are quite comparable, in terms of tool classes represented and their proportions (Table 4). Bifacial tools, bifacial roughouts, irregular denticulates, notches, perforators, points, retouched pieces and utilized pieces are present in both units. Units 1 and 4 differ mainly in that there are more bifacial roughouts and preforms in Unit 1 than in Unit 4. In contrast, Units 2 and 3 do not have as many different kinds of tools. All of the tools in Unit 2 are roughouts, there are no finished tools. Unit 3 has barely any tools, just two roughouts and a notch.

**Table 4:** Counts and percentages of tool types in L20 Units 1–4.

	Unit 1		Unit 2		Unit 3		Unit 4	
Item	Count	%	Count	%	Count	%	Count	%
Bangle (Frag)	0	0	0	-	0	-	1	2.17
Bifacial Knife	1	3.7	0	-	0	-	0	0
Preform (Knife)	1	3.7	0	-	0	-	0	0
Roughout (Bifacial tool)	2	7.41	0	-	0	-	1	2.17
Limestone Mining tool	0	0	0	-	0	-	1	2.17
Roughout-Bifacially prepared edge	3	11.11	4	-	0	-	3	6.52
Roughout- Bifacially prepared edge (Frag)	3	11.11	5	-	0	-	1	2.17
Roughout-Indeterminate purpose	0	0	1	-	2	-	2	4.35
Roughout-Indeterminate purpose (Frag)	1	3.7	1	-	0	-	0	0
Scraper (Frag)	0	0	0	-	0	-	2	4.35
Irregular denticulate	0	0	0	-	0	-	1	2.17
Iregular denticulate (Frag)	1	3.7	0	-	0	-	0	0
Notch	5	18.52	0	-	1	-	7	15.22
Notch (Frag)	1	3.7	0	-	0	-	0	0
Notch on a Truncation	0	0	0	-	0	-	1	2.17
Truncation	1	3.7	0	-	0	-	0	0
Perforator	1	3.7	0	-	0	-	5	10.87
Point	1	3.7	0	-	0	-	1	2.17
Retouched Piece	2	7.41	0	-	0	-	7	15.22
Retouched Piece (Frag)	0	0	0	-	0	-	2	4.35
Ind Tool Frag	2	7.41	0	-	0	-	6	13.04
Utl Blade/ Blade segment	2	7.41	0	-	0	-	1	2.17
Utl Flake	0	0	0	-	0	-	4	8.7
TOTAL	27	99.98	11	-	3	-	46	99.98

Artifact size was the final aspect of unit assemblages analyzed, and this attribute also shows very clear differences. Unit 3 has significantly more large and very large pieces (Table 5).

**Table 5:** Counts and percentages of artifact sizes across all four units.

	Unit 1		Unit 2		Unit 3		Unit 4	
Size data	Count	%	Count	%	Count	%	Count	%
Very small 1–1.5 cm	428	20.26	44	8.48	19	3.13	107	6.6
Small 1.5–5 cm	1555	73.59	374	72.06	364	59.97	1145	70.59
Medium 5–10 cm	119	5.63	88	16.96	168	27.68	327	20.16
Large 10–20 cm	11	0.52	13	2.5	52	8.57	42	2.59
Very large 20+ cm	0	0	0	0	4	0.66	1	0.06
TOTAL	2113	100	519	100	607	100.01	1622	100

#### Summary & interpretation of the units

The activities that resulted in the artifact assemblages for each unit can be summarized as follows: The assemblage in Unit 1 probably resulted from a variety of knapping episodes over a long time span, including blade production and especially bifacial tool production, since Unit 1 had many very small artifacts (chips), many thinning flakes, pressure flakes, a high percentage of roughouts, some blade cores, and a variety of tools. Moreover, the Unit 1 surface collection was located in the shade of a natural rock outcrop (Fig 23), and this rock outcrop was surrounded by stratified accumulations of artifacts, as could be seen in the profile of a looters hole there (Fig 7 bottom). It is very clear that the remains in Unit 1 resulted from both blade production and bifacial tool production, but that there was more focus on bifacial tool reduction and fine tool finishing in that area than in the others. This is significant because it shows that at least some tools were completely finished at Wadi el-Sheikh, and that the activities at this mining site were not limited to raw material extraction or initial preparation of cores and tools.

Unit 2 was located right on the edge of an opencast mine (Fig 19) and likely represents initial preparation of raw materials, including bifacial preparation. The Unit 2 assemblage included initially tested cores, and the only tools found there were roughouts. Furthermore, there was no clear indication of continuing stratigraphy after removal of the surface material, and the density of artifacts was relatively low.

The Unit 3 assemblage consisted of large artifacts, initially tested pieces, few tools, and a lot of angular debris. Most of the artifacts were silicified limestone, not chert. Considering that the artifacts formed a rather concentrated group, were deposited mainly on the surface with little apparent stratigraphy, and comprised a limited range of debitage and tool types, this assemblage is probably a dump of artifacts from the raw material extraction process.

**Fig 33:**
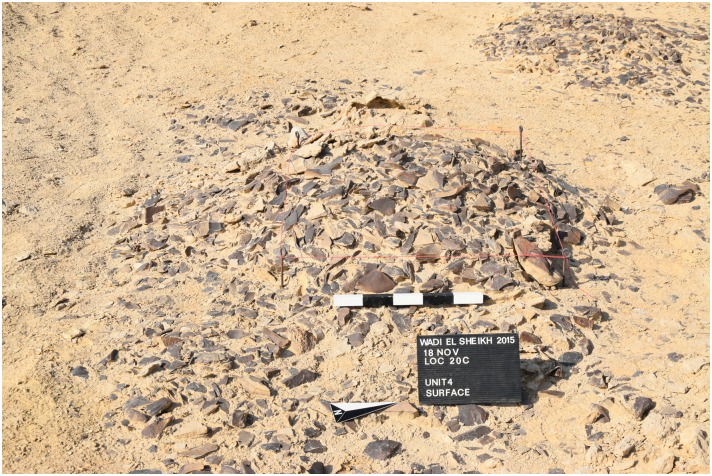
Unit 4 at L20C. Unit 4 before excavation. The unit was placed in a concentration of lithic material, and removal of the surface layer produced more than 1600 pieces of chert including 45 cores, which primarily result from blade production (Photo M. Klaunzer).

The remains in Unit 4 (Fig 33) resulted mainly from blade production, as evidenced by the many blade cores and the high percentage of blades. Unit 4 was located among a series of large dense artifact concentrations in front of a natural rock shelter (much like Unit 1), and the deposit was stratified, since more materials could be seen continuing in the layer below, after removal of the surface artifacts. Since there is also some evidence of bifacial artifact production in the unit (such as the bangle fragment), and the deposit is not confined only to the surface but continues below the surface, the assemblage in this unit probably results from a variety of activities over a long time span, but with more of a focus on blade production.

The assemblages in Units 1–4 demonstrate a number of significant points about site use. The different foci of the unit assemblages—raw material extraction (U3), initial processing (U2), blade production (U4), and bifacial tool production/finishing (U1)—indicate that the site did not result from a series of similar activities taking place across the landscape, but that production was likely segmented. Chronological differences in activities could account for some of the variation between the units. However, at least some of the differences must be the result of segmented production, since finishing stages of production and blade production were evident in Units 1 and 4, respectively, but these units were well separated from the extraction areas, and did not contain large amounts of material from initial processing. This segmentation implies both organization and a larger scale of workers.

The existence of stratified remains in Units 1 and 4 was apparent from the presence of more artifacts in the layer below, after the removal of surface materials, and in the profile seen in looters' pits. The stratified deposits show that the site was used over time, and the pattern of stratified remains existing near rock shelters (Units 1 & 4) rather than in more open areas (Units 2 & 3) shows that some practical aspects of site use did not change. The presence of both bifacial tool production and blade production in Units 1 & 4, but in different proportions, could indicate either that the main focus of production varied in different parts of the site, or that it varied over time, which is a question that will be explored in the future.

## The logistics of resource acquisition, manufacturing technology and ancient Egyptian economy

The Wadi el-Sheikh is one of the largest, most continuous, complex and best preserved chert exploitation areas in the Near East and Africa, possibly in world archaeology. Together with the intensive archaeological study of prehistoric and Pharaonic societies of the Egyptian Nile Valley over the past 120 years, it offers significant evidence for ancient Egypt's raw material acquisition and manufacturing technologies, work organization, distribution and transport systems, both in the socio-economic context of prehistoric non-state and Pharaonic complex state society. The latter is especially pertinent for archaeologists throughout the world: the Wadi el-Sheikh mining activities cover a long period of time, from the Neolithic (ca. 5,000–4,000 B.C.E.) until the late New Kingdom (ca. 12th century B.C.E.), and the presence of tools from the Middle Palaeolithic (ca. 175,000–50,000 B.P.) indicates that there could be even earlier chert extraction in the area such as that found for the Middle and Upper Palaeolithic in other parts of Egypt (see below). During all this time Nile Valley societies underwent the whole range of social transformations from mobile hunter-gatherer societies via emerging chiefdoms to a highly centralized territorial state society, each with their own specific raw material acquisition strategies and economic systems. And yet, what remained the same over all those hundreds of thousands of years was the material they were keen to exploit, i.e. chert, and the region they decided to do so: Wadi el-Sheikh. This area therefore offers unique insights into the technological and organizational aspects of chert exploitation at a certain period of time, and over time.

To understand the acquisition of this material and the precise manufacturing technologies, patterns of distribution, division and organization of labor, i.e. their whole *chaîne opératoire* [29, 30, 31], together with their distribution and consumption in the socio-economic context of their ultimate functions, means to understand a very large segment of material culture and a whole economic sector of ancient Egyptian society.

However, despite the long duration of Egyptian civilization, only a small number of other mining areas where chert was systematically mined underground are known along the Egyptian Nile Valley (Table 1). These are the Middle and Upper Palaeolithic mines at Taramsa Hill and at Nazlet Khater-4 in southern Egypt investigated by Belgian missions in the 1980-90s [32, 33,34], a site near Hierakonpolis [35], quarries near Tell el-Amarna which may date to the New Kingdom period [36] and the wider Wadi Araba region (Galala North/Wadi Sannur/Warag, Ain Barda and Wadi Umm Nikhaybar quarries) in northern Middle Egypt currently being investigated by a French mission. The latter at Galala North have been dated primarily to the Early Dynastic and Old Kingdom periods [37,38]. Considering the wide use of stone tools in prehistoric and Pharaonic culture through the late 2nd Millennium B.C.E., there are obvious gaps in the representation of mining sites where the raw material of these periods was exploited. This is where the Wadi el-Sheikh has its main potential because it has the longest time depth and greatest intensity of chert exploitation of any site in Egypt dating at least from the Neolithic until the New Kingdom. Notably, the Wadi hence offers great insights not only into the long transitional period when society transformed from non-state societies living in independent farming villages along the Nile via chiefdom societies, and finally the early territorial state. But it also allows for observing how chert exploitation strategies changed relative to the political expansions and contractions during Pharaonic history. Research at Wadi el-Sheikh provides the opportunity to show how the scale of mining and tool production changed over time and to reveal details of differences in the organization of labor and the logistics involved that would relate to these significant socio-political transformations in the Nile Valley.

Together with a few other ancient civilizations such as Mesopotamia, China, Maya, and Classical Antiquity, Pharaonic Egypt is in the fortunate situation of being able to draw detailed information from written sources. For the historical period, there is indeed a large body of Egyptological literature that deals with economic aspects of resource acquisition and especially formal, state-organized expeditions to remote areas including the Eastern Desert and Sinai regions for the procurement of raw materials such as copper, gold and stone for construction. This is because in many well-documented cases, the expedition leaders were state officials who left behind graffiti and decorous hieroglyphic inscriptions commemorating their visits to the mines and quarries [39, 40, 41]. For example, there are more than 30 such inscriptions from the Old Kingdom, which inform on the personnel and duration of such formal expeditions. From these we know that they could last between one and several months and that the officials were equipped with dozens or sometimes hundreds of workmen, specialists, and support personnel including bakers, shoe makers and security. While no such formal inscriptions have as yet been found at the Wadi, and none of the inscriptions published to date deal specifically with chert mining, they still allow us to better contextualize the archaeological evidence from the Wadi itself. For example, from the vast amount of material that has been moved in the area of L20 or the extent and number of the mining shafts in L5 and L11, it is clear that sizable teams of workmen must have been employed to accomplish this work. Evidence for compartmentalized stone-built structures in these areas (as in Fig 22) may be interpreted as barracks where the workmen lived during the expedition.

Another very important issue to consider in this context is the water supply, and the possible transport thereof, as there does not seem to be a natural water source in the Wadi. Previous studies on the Abu Ballas trail in the Western Desert [42,43], an important caravan route during the 2nd Millennium B.C.E, are very useful in this regard as the authors have provided specific calculations for the water supply of donkey caravans in arid climates. We assume that donkeys were also the main transport animals for the Wadi because the domesticated donkey has been in evidence in Egypt at least since the early 4th Millennium B.C.E. [44,45], and the camel does not feature until the 1st Millennium B.C.E. It is possible that, like on the Abu Ballas trail, water supplies, stored in ceramic containers and brought by preparatory expeditions, had been buried in the ground along the route. But from the calculations alone, it is clear that sending a mining expedition to the Wadi was as challenging in ancient times as it is today: based on a mining expedition of five days duration with only two workers and two donkeys, more than 300 liters of water, plus food and equipment, needed to be carried to the Wadi for the workmen to engage in hard labor at the mines. The evidence at L5, 11 and 20, measured especially on the basis of the amount of chert extracted and processed on the spot as well as by the enormous dimensions of spoil heaps accumulated nearby, suggests that the labor was likely done by far more than just two persons and over more than five days at a time. For example, the amount removed from just one underground exploitation chamber at L20C has been calculated at 208 tons of excavated material. It is also possible to calculate the extracted volume of a single mining shaft of 1.5 m diameter and 5 m depth at approximately 8.8 m^3^, which corresponds to about 22 tons of chert and limestone (this calculation only applies to the shaft itself not counting any additional underground chambers). In an area like L11, where we estimate a number of 40 such shafts, the amount would add up to about 352 m^3^ or 880 tons of manually excavated rock.

Additionally, our preliminary work has already indicated a degree of labor division. For example in L20, as discussed above, we have observed variability in the lithics assemblages of distinct areas where raw material extraction, initial processing, blade production and bifacial tool production/finishing have mainly taken place in different areas, which may be interpreted as evidence for the division of labor. This would suggest that the labor force was not only comprised of unskilled laborers but possibly also involved workmen and specialists engaged in specific activities. It would therefore be reasonable to assume that one such mining expedition may have required a whole gang of different workmen engaged in hard labor over several weeks and that there would have been a degree of internal professional hierarchy among the workmen. The more workmen were involved, the more material could be extracted, but the more supplies, and especially food and water, had to be brought to the Wadi. Interestingly, L20, which is probably one of the largest and most intensively exploited mining localities recorded by us until now, dates to the early Pharaonic period, i.e. Early Dynastic and Old Kingdom. This is a most formative phase when the incipient territorial state was in the process of significant socio-political developments towards complex society and internal administrative and economic restructuring of the country that eventually resulted in the highly centralized Pharaonic state of Old Kingdom Egypt, capable of building nothing less than the great pyramids of Giza. L20 is probably contemporary with the chert/silex mines at Galala North/Wadi Sannur mentioned above. In combination, they document both the enormous demand for chert/silex during this first phase of intensified centralized state economy as well as the effort expenditure invested in exploiting this raw material at a very large scale. This is even more astounding considering the massive human and material resources that the central administration and construction project management had concentrated in the area of the state capital Memphis where the pyramids were being built at the same time.

Importantly, however, although chert acquisition must be regarded a significant economic activity in Pharaonic civilization given the wide use of chert in almost every area of life and by every group in the social hierarchy of Pharaonic society, it has received almost no attention in the Egyptological literature so far. In the long term, our research will therefore add a new body of data valuable for archaeologists and Egyptologists alike based on empirical archaeological evidence obtained from Wadi el-Sheikh.

## Summary and outlook

Whilst our research at the Wadi is still in its early days, the empirical data that we have already gained over three relatively brief seasons of fieldwork are very significant and most encouraging as they underline the importance of Wadi el-Sheikh for the archaeology of ancient chert exploitation and for the archaeology of ancient Egypt. Our surveys have shown that these mining areas are dense, vast and stretched over a very large region of yet unknown size. Thus, extensive and intensive surveys at the Wadi will continue. More focused study and excavation of select areas, especially at L20, have already shown that it is important to not only comprehend the Wadi as a whole and to investigate the size and chronological scope of the different mining areas, but also to explore the duration, quantity and volume of material extracted and processed on the spot, and thereby to reconstruct the possible organization and expenditure of labor invested. Future investigations will continue in a number of other areas with intensive chert mining and will include focused excavations of building structures and camp sites in order to achieve high quality archaeological data on the chronological time frame, assisted by chronometric analyses, and the specific living conditions of the ancient miners. Furthermore, in cooperation with a number of other field projects in Egypt, future research will include scientific analysis of chert artifacts found in Nile Valley settlements and cemeteries so as to precisely determine the geographical distribution of the Wadi el-Sheikh chert in time and over time, and to contextualize the social and economic impact of the ancient activities in this unique and long-lasting site.
